# Effects of ezetimibe on cholesterol metabolism in HIV-infected patients with protease inhibitor-associated dyslipidemia: a single-arm intervention trial

**DOI:** 10.1186/1471-2334-14-497

**Published:** 2014-09-11

**Authors:** Pere Leyes, Esteban Martínez, María Larrousse, Montserrat Cofán, Joan Trabal, Ana María Pérez-Heras, María T Forga, Emilio Ros

**Affiliations:** Endocrinology and Nutrition Service, Hospital Clínic, Universitat de Barcelona, Villarroel 170, Barcelona, 08036 Spain; Infectious Diseases Unit, Institut d’Investigacions Biomèdiques August Pi Sunyer (IDIBAPS), Hospital Clínic, Villarroel 170, Barcelona, 08036 Spain; Lipid Clinic, IDIBAPS, Hospital Clínic, Villarroel 170, Barcelona, 08036 Spain; CIBER Fisiopatología de la Obesidad y Nutrición (CIBERobn), Instituto de Salud Carlos III (ISCIII), Madrid, Spain; Fundació Clinic per la Recerca Biomèdica, Hospital Clínic, Villarroel 170, Barcelona, 08036 Spain

**Keywords:** HIV infection, Cholesterol, Phytosterols, Ezetimibe, Protease inhibitors

## Abstract

**Background:**

The effects of ezetimibe on cholesterol metabolism in HIV-infected patients receiving boosted protease inhibitors have not been thoroughly assessed. The aim of this study was to assess cholesterol homeostasis in patients with PI associated dyslipidemia and its relationship with the response to treatment with the cholesterol-absorption inhibitor ezetimibe.

**Methods:**

Fifteen patients with ritonavir-boosted PI-containig therapy and LDL-cholesterol > 3.36 mmol/L (>130 mg/dL) were assessed at baseline and after an 8-week course of ezetimibe 10 mg/d. Serum non-cholesterol sterols were measured at each visit as markers of cholesterol synthesis and absorption. Total-, LDL-, and HDL-cholesterol triglycerides, apolipoproteins A1 and B, high sensitivity C-reactive protein, CD4 cells and HIV-1 RNA were also measured.

**Results:**

Ezetimibe treatment was well tolerated in all patients and resulted in significant reductions in total cholesterol (-11.4%, p = .002), LDL-cholesterol (-20.4%, p = .003), non-HDL-cholesterol (-13.4%, p = .002) and apolipoprotein B (-9.1%, p = .021).

Treatment with ezetimibe was associated with decreased cholesterol absorption markers (campesterol-to-cholesterol ratio -43.0%, p = .001; sitosterol-to-cholesterol ratio -41.9%, p = .001) and increased synthesis markers (lathosterol-to-cholesterol ratio 53.2%, p = .005). Baseline absorption or synthesis markers were unrelated to the response to treatment. CD4 cell count and plasma HIV-1 RNA remained unchanged.

**Conclusions:**

The level of cholesterol absorption or synthesis does not appear to be a major determinant of the responsiveness to ezetimibe in patients on ritonavir-boosted PI-containing therapy.

**Trial registration:**

EudraCT: 2006-006156-36

**Electronic supplementary material:**

The online version of this article (doi:10.1186/1471-2334-14-497) contains supplementary material, which is available to authorized users.

## Background

Abnormalities of lipid metabolism are common complications of HIV therapy, particularly with protease inhibitors (PI) [1]. Antiretroviral treatment exposure is an independent risk factor for cardiovascular disease in HIV-infected patients [2], which is particularly linked to PI exposure and relates in part to lipid alterations [3]. This risk adds to conventional risk factors, particularly smoking, which is highly prevalent among seropositive patients [4]. In addition, HIV-associated inflammation and vascular dysfunction may also lead to increased cardiovascular morbidity despite successful antiretroviral therapy (ART) [5].

Although statins are considered the drugs of choice for attaining LDL-cholesterol targets in HIV-infected patients [6], some statins may display clinically significant interactions with common antiretroviral drugs through inhibition of CYP3A metabolic pathways [7]. Furthermore statin therapy in HIV-infected patients has a lipid-lowering efficacy lower than expected, thus patients may require high doses or additional cholesterol-lowering agents [8]. Ezetimibe is a lipid lowering agent that reversibly inhibits intestinal cholesterol absorption without interfering with hepatic P-450 enzymes [9], thus showing a low potential for drug interactions compared with currently available statins. As PI increase cholesterol synthesis through activation of SREBP regulated pathways [10], it is unknown whether the use of a cholesterol-absorption inhibitor in HIV-infected patients treated with PI induces different changes in cholesterol metabolism than those reported in non-HIV infected dyslipidemic patients.

Small amounts of non-cholesterol sterols circulate in blood and can be used as reliable biomarkers of cholesterol metabolism [11]. Plasma cholesterol precursors such as lathosterol are markers of cholesterol synthesis and, when expressed as ratios to cholesterol, show a good correlation with synthesis rates measured by isotope kinetic techniques [12]. Plant sterols or phytosterols are absorbed in the small bowel via the same receptor involved in cholesterol absorption (NPC1L1) [13]. Therefore, plasma levels of the main phytosterols, campesterol and sitosterol, can be used as indicators of cholesterol absorption [8, 14].

In general population with hypercholesterolemia, treatment with ezetimibe resulted in decreased LDL-cholesterol by -17 to -20% [15–17]. A reduction in campesterol/cholesterol and sitosterol/cholesterol ratios (-41 and -34%, respectively), and an increase in lathosterol/cholesterol ratio (+72%) has also been reported [15, 17, 18].

The aim of this study was to assess cholesterol homeostasis in patients with PI-associated dyslipidemia and its relationship with the response to treatment with the cholesterol-absorption inhibitor ezetimibe.

## Methods

### Patients

HIV-infected adults (≥18 years) receiving stable ritonavir-boosted PI-containing ART, with plasma HIV-1 RNA below 50 copies/ml at least for the previous 6 months, and presenting with plasma LDL-cholesterol levels between 3.36 and 4.91 mmol/l (130–190 mg/dL) without statin therapy were considered eligible for study. Exclusion criteria were: hypersensitivity to ezetimibe, consumption of phytosterol-enriched functional foods within one month before study entry, prior history of cardiovascular disease, secondary hypercholesterolemia, renal insufficiency (defined by a glomerular filtration rate (MDRD) <60 mL/min/1.73 m^2^), increased transaminase levels (defined by 5 times above the upper level of normality), concomitant treatment for hepatitis virus coinfections, diabetes mellitus, active illicit drug or alcohol abuse, AIDS-defining opportunistic infection within 3 months prior to study entry, any acute illness within one month prior to study entry, and pregnancy or breastfeeding. Criteria for patient withdrawal from the study were any changes in antiretroviral regimen during the study period, including dose changes or drug discontinuation.

The study was approved by the institutional review board of Hospital Clinic de Barcelona, and conducted according to good practice guidelines. All patients provided written informed consent before study entry.

### Study design

In this open-label intervention study, participants received ezetimibe 10 mg/d during 8 weeks. At study entry, all patients received written recommendations to ensure avoidance of phytosterol supplements during the study period. Patients were scheduled for visits at baseline and week 8. At each visit 3-day food records were obtained and the nutrient composition of the diet was assessed by using Food Process-Plus software (version 8.0). Spent boxes of medication were collected to assess compliance. Anthropometric measurements were made by standard methods. Waist circumference was measured in the upright position half way between the lower subcostal margin and the iliac crest. Previous ART was recorded as time of exposure to combination ART (cART) as well as to thymidine and non-thymidine analogues (TA) Reverse Transcriptase Inhibitors (RTI), PI, and non-Nucleoside Reverse Transcriptase Inhibitors (NNRTI) families.

### Laboratory methods

Fasting blood samples were obtained at baseline and at the end of the treatment period. Except for standard biochemistry and blood-cell analyses, serum samples were stored at -80°C and analyzed at the end of the study. Total cholesterol, triglycerides, and HDL-cholesterol [after precipitation of apolipoprotein (apo) B–containing lipoproteins] were determined by standard enzymatic methods in an automatic analyzer DDPPII Hitachi (Roche, Basle, Switzerland), using specific reagents (Boehringer-Mannheim, Mannheim, Germany). LDL-cholesterol was calculated by using the Friedewald equation. ApoAl and apoB were determined by using turbidimetry. Concentrations of variables reflecting glucose control and kidney and liver function, i.e., fasting glucose, total bilirubin, aspartate aminotransferase, alanine transaminase, alkaline phosphatase, γ-glutamyl transpeptidase, and creatinine were determined on the automatic analyzer. Hematological variables (total and differential white blood cell count, red blood cell count, hemoglobin concentration, hematocrit, mean corpuscular volume, and platelet count and volume) were determined on a Coulter counter.

Non-cholesterol sterols and high sensitivity C-reactive protein (CRP) were determined in frozen samples of whole serum. Plant sterols (campesterol and sitosterol) and the cholesterol precursor lathosterol were analyzed by gas chromatography as previously described [19]. Briefly, epicoprostanol (2 μg) was added to serum (0.1 ml) as internal standard. After alkaline hydrolysis, extraction, and derivatization to trimethylsilyl ethers, the sterols were quantified by gas chromatography on a 30 m nonpolar capillary column (TRB-Esterol; Teknokroma, Barcelona, Spain) with a Perkin-Elmer GC Autosystem (Perkin-Elmer, Norwalk, CT, USA). Plant sterol and precursors concentrations are expressed as ratios to cholesterol (μmol/mmol cholesterol). Inter- and intra-assay CVs were 5.0% and 3.2% for lathosterol, 1.9% and 1.6% for campesterol, and 2.0% and 1.8% for sitosterol, respectively. CRP was determined by turbidimetry. The intra-assay coefficient of variation for CRP was 3.1%.

The number of CD4 T lymphocytes was measured by flow cytometry. Plasma HIV-1 RNA was measured by a PCR assay (Amplicor HIV Monitor, Roche Diagnostics Systems, Branchburg, New Jersey, USA; lower limit of detection 50 copies/mL).

### Statistical analyses

The primary efficacy variable was the change in serum LDL-cholesterol. Key secondary variables were changes in other lipids, non-cholesterol sterols, and CRP. Variables were described as medians and interquartile ranges. Outlier values of CRP (1.5 times the interquartilic range above the median) were excluded from the analysis of CRP response. A sample size of 15 evaluable patients was considered necessary to detect a decrease of at least 20% in LDL-cholesterol, according to previous data [20], with an 80% power and a significance level of 0.05, using a two-sided test. We used the Wilcoxon rank test for within-group comparisons and the Spearman’s rho test for correlation between continuous variables. Analyses were performed using SPSS software version 16.0.

## Results

### Baseline characteristics

Fifteen patients (9 men and 6 women, mean age 53 years) were included and eligible for analysis. Eight patients were active smokers. Median ART exposure was 504 w (range 228–581). Median exposures to specific therapies were: non-TA RTI, 449 w; TA RTI, 303 w; PI, 350 w, and NNRTI, 32 w. Intake of selected nutrients did not change between time points. The intervention had no effect on body weight or waist circumference (Table 1).

**Table 1:** **Baseline data and outcome variables in 15 patients treated with ezetimibe for 8 weeks**

Variables	Baseline	Treatment	% change	P*
Dietary assessment				
Total calories (kj/d)	8037 [6728–9033]	7657 [6841–11167]	-4.7%	.55
Total fat (g/d)	93 [76–105]	86 [83–115]	-7.5%	.53
Saturated fatty acids (g/d)	26 [17–31]	24 [19–32]	-7.7%	.94
Cholesterol (mg/d)	254 [223–340]	294 [247–346]	15.7	.81
Phytosterols (mg/d)	184 [141–211]	156 [129–193]	-15.2%	.09
Body weight (kg)	73.0 [61.2-80.1]	73.1 [63.9-80.8]	0.1%	1
Waist circumference (cm)	92.5 [85.7-96]	95.0 [88–100]	2.7%	.55
Lipids and apos				
Total cholesterol (mmol/L)	5.87 [5.46-6.54]	5.2 [4.89-5.61]	-11.4%	.002
LDL-cholesterol (mmol/L)	3.93 [3.46-4.27]	3.13 [2.95-3.44]	-20.4%	.003
nonHDL-cholesterol (mmol/L)	4.63 [4.29-5.30]	4.01 [3.59-4.42]	-13.4%	.002
HDL-cholesterol (mmol/L)	1.21 [1.11-1.47]	1.19 [1.03-1.34]	-2.1%	.11
Triglycerides (mmol/L)	1.64 [1.15-2.04]	1.42 [1.15-2.29]	-13.1%	.46
Apo A1 (g/L)	1.47 [1.39-1.53]	1.54 [1.44-1.59]	4.7%	1
Apo B (g/L)	1.21 [1.09-1.42]	1.10 [0.95-1.24]	-9.1%	.02
C-reactive protein (mg/L)	1.2 [0.4-1.6]	0.6 [0.5-3.5]	-50%	.61
Plasma viral load (copies/mL)	49 [49–49]	49 [49–49]	0%	.65
CD4 cell count (cells/μL)	503 [353–802]	499 [323–794]	-0.8%	.65
Lathosterol (μmol/L)	7.08 [5.19-9.43]	9.97 [6.68-12.67]	40.9%	.015
Lathosterol/cholesterol	1.26 [0.84-1.54]	1.93 [1.37-2.40]	53.2%	.005
Campesterol (μmol/L)	27.78 [20.22-38.95]	14.78 [10.72-18.51]	-46.8%	.001
Campesterol/cholesterol	4.65 [3.23-6.36]	2.62 [1.98-3.53]	-43.6%	.001
Sitosterol (μmol/L)	22.96 [20.34-33.25]	14.77 [10.96-19.52]	-35.7%	.001
Sitosterol/cholesterol	4.46 [3.70-6.09]	2.59 [2.17-3.76]	-41.9%	.001

Results are expressed as medians [IQR]. *Wilcoxon Rank test.

### Efficacy

**Figure 1:**
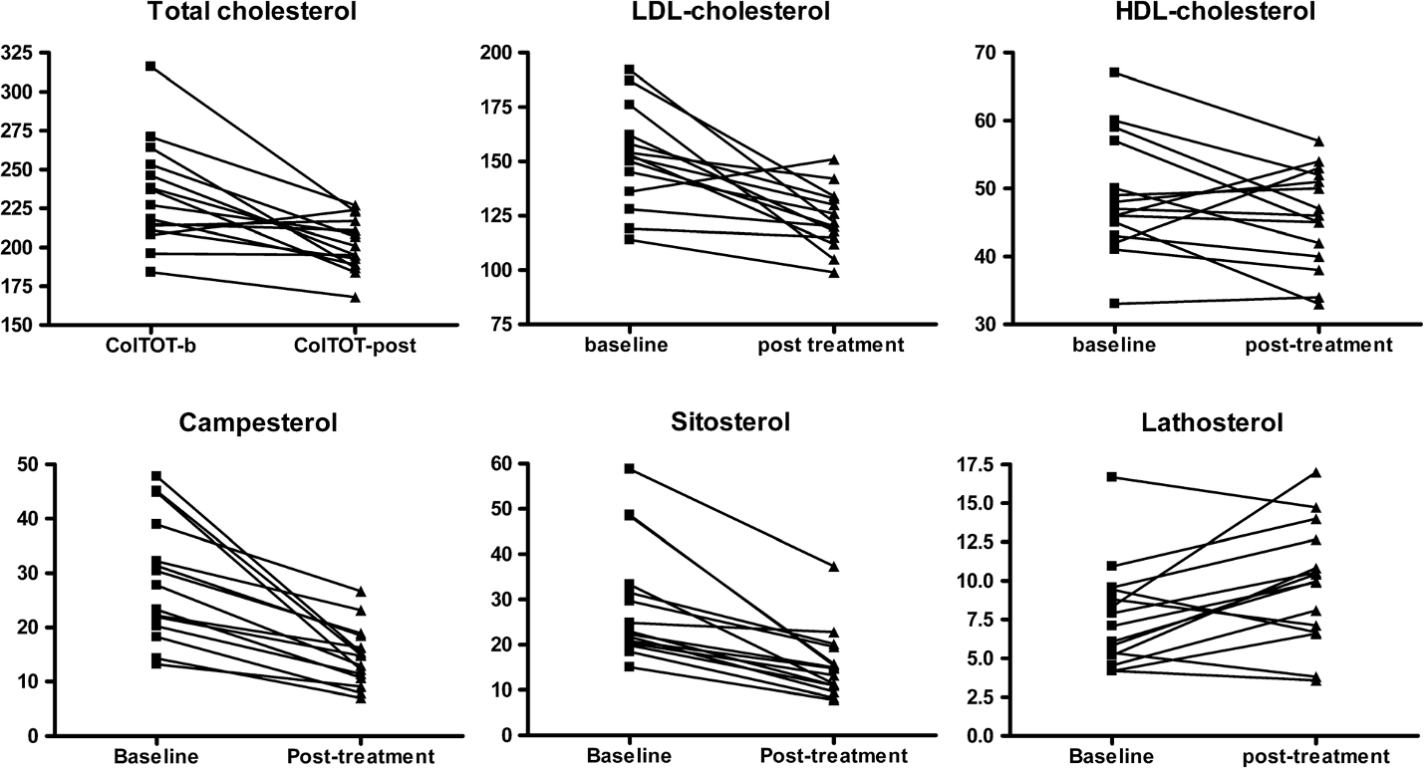
**Individual changes in total cholesterol, lipoprotein cholesterol and non-cholesterol sterols after ezetimibe treatment.**

After 8 weeks of ezetimibe treatment, significant changes from baseline were observed for total cholesterol (-0.67 mmol/L; p = .002), LDL-cholesterol (-0.80 mmol/L; p = .003), non-HDL-cholesterol (-0.62 mmol/L; p = .002) and apoB (-0.11 g/L; p = .021). No significant changes were observed for HDL-cholesterol, triglycerides, apoA1 or CRP (Table 1). Total cholesterol and LDL-cholesterol responses were consistent in most patients (Figure 1).

Treatment with ezetimibe was associated with a reduction in plasma campesterol and sitosterol concentrations in every participant, which confirms participants’ compliance with the study drug. Similar results were observed for their ratios to cholesterol. Conversely, plasma lathosterol increased in most treated subjects (Table 1, Figure 1).

Concerning safety, CD4 cell count and plasma HIV-1 RNA remained unchanged. No changes in routine biochemistry or hematologic parameters were observed.

### Relationship between markers of cholesterol metabolism and response to treatment

Baseline campesterol- and sitosterol-to-cholesterol ratios (as markers of absorption) and the lathosterol-to-cholesterol ratio (as a marker of synthesis) were not associated with the cholesterol lowering response to ezetimibe, as judged by the lack of correlation between these variables and changes in LDL-cholesterol after treatment. No correlation was observed either between baseline lipid levels and these markers.

Baseline campesterol- and sitosterol-to-cholesterol ratios were highly correlated between them (r = .932; p < .001). Although lathosterol-to-cholesterol ratio increased as expected in response to cholesterol absorption inhibition, the changes were unrelated to those of either campesterol or sitosterol. Changes in CRP did not correlate with changes in plasma lipids or non-cholesterol sterols.

Previous antiretroviral exposure (total cART or subfamilies: TA- and non-TA-RTI, PI, NNRTI) did not show any correlation with markers of cholesterol absorption or synthesis. No association existed between antiretroviral exposure and baseline lipid levels either.

## Discussion

In this study we confirmed that 8 weeks of ezetimibe monotherapy was effective in reducing total and LDL-cholesterol in HIV patients receiving boosted-PI containing ART. The reduction in LDL-cholesterol levels (-20.4%) was in the expected range of responses reported in studies of ezetimbe monotherapy in the general population with dyslipidemia (-16% to -20%) [15–17], as well as in HIV-infected patients [21]. According to our data, seropositive patients receiving boosted-PI do not necessarily have a weaker response to ezetimibe, as suggested by *Wohl et al.* [20]. These authors found a poor response to ezetimibe (-5.3% LDL-cholesterol reduction) in HIV patients on triple therapy, but their mean baseline LDL-cholesterol (3.31 mmol/L) was lower than that in our population, which could account, at least in part, for the differences observed.

However, despite its efficacy in reducing serum cholesterol levels, the effectiveness of ezetimibe in reducing major cardiovascular events is yet to be proven. There is an ongoing clinical trial (IMPROVE-IT; ClinicalTrials.gov identifier NCT00202878) addressing this issue.

In our study ezetimibe resulted in a reduction in cholesterol absorption markers and an increase in cholesterol synthesis markers, which were in the range of the changes reported in non-HIV dyslipidemic subjects [15, 17, 18], suggesting that PI-containing ART has no major effect on cholesterol absorption or synthesis. However, the level of cholesterol absorption does not appear to be a major determinant of the responsiveness to ezetimibe, as shown by the lack of correlation between cholesterol absorption or synthesis markers and baseline lipid levels or their response to therapy. Other studies in non-HIV hypercholesterolemic subjects have produced similar results [22, 23]. In one study, only a weak inverse correlation (r: -0.17) was found between baseline campesterol-to-lathosterol ratio and the response to ezetimibe [18]. In our study, neither baseline sitosterol- nor campesterol-to-lathosterol ratios correlated with response to therapy (data not shown). It is possible that factors downstream the primary site of action of ezetimibe could be major determinants of response.

In our study CRP decreased non-significantly after ezetimibe treatment. The great intra- and inter-individual variability observed with this marker makes it difficult to detect changes, at least in small studies. In a meta-analysis assessing the contribution of LDL-dependent effects of cholesterol-lowering therapies to changes in CRP, most of the CRP reduction was related to LDL changes [24]. As noted above, the small sample size is one limitation of our study, particularly for secondary outcomes. However, the study population was quite homogeneous for antiretroviral therapy and viral control, ruling out any potential influence of antiretroviral changes or uncontrolled HIV replication on the results.

## Conclusions

The pharmacological inhibition of cholesterol absorption by ezetimibe is followed by a compensatory increase in cholesterol synthesis. The level of cholesterol absorption or synthesis prior to therapy does not appear to be a major determinant of the responsiveness to ezetimibe. Our findings in HIV patients receiving ritonavir-boosted PI-containing therapy are similar to those reported in non-HIV hypercholesterolemic subjects, suggesting that therapy with protease inhibitors by itself does not substantially affect cholesterol homeostasis.

## Authors’ original submitted files for images

Below are the links to the authors’ original submitted files for images. Authors’ original file for figure 1

## Abbreviations

ART: Antiretroviral therapy
cART: Combination ART
CRP: C-reactive protein
NPC1L1: Niemann-Pick C1-Like 1
PI: Protease inhibitors
RTI: Reverse Transcriptase Inhibitors
SREBP: Sterol regulatory element-binding protein
TA: Thymidine analogues.
