# Supplementary figures and images for: Effects of ezetimibe on cholesterol metabolism in HIV-infected patients with protease inhibitor-associated dyslipidemia: a single-arm intervention trial
Source: BMC Infect Dis. 2014 Sep 11;14:497. doi: 10.1186/1471-2334-14-497 (PMC4169814; doi:10.1186/1471-2334-14-497)

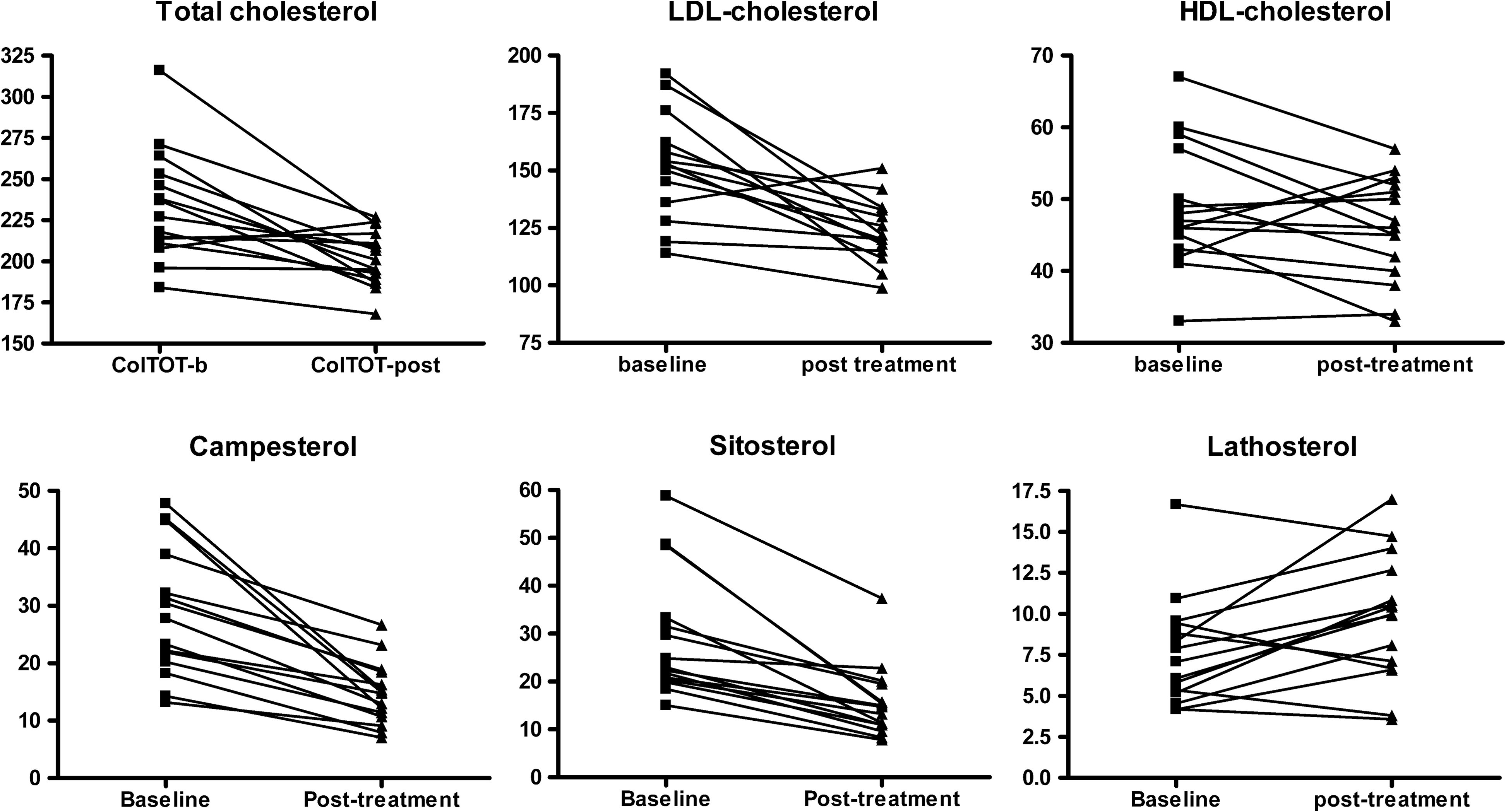

Supplement: Supplementary file 1 — Authors’ original file for figure 1 [file 12879_2014_3802_MOESM1_ESM.tif]
